# Innocentism: Preferring the Innocent Over the Culpable

**DOI:** 10.1007/s10790-022-09926-1

**Published:** 2022-12-17

**Authors:** Nico Dario Müller

**Affiliations:** Philosophical Seminar (Department of Arts, Media, Philosophy), University of Basel, Steinengraben 5, 4051 Basel, Switzerland

## Introduction

In a burning building, would you rather save a dog or a war criminal? If you would rather save the dog, you might be an innocentist. By the neologism “innocentism,” I refer to the view that the morally innocent deserve greater moral concern than the morally culpable, other things being equal. More specifically, I mean the view that moral deservingness of concern decreases as moral culpability increases. An innocentist cares more about the innocent than the culpable, assigns greater weight to their interests. And the more numerous and serious someone’s moral transgressions are, the less the innocentist cares about their weal and woe.

While the term “innocentism” is new, the view itself is not unheard of. Kagan, for one, brings it up as an intuitive counterexample to the Principle of Equal Consideration of Interests.^1^ Jaquet also mentions it, noting its widespread intuitive support.^2^ Regan discusses and rejects a radical cousin of innocentism, the “innocence principle,” according to which the innocent must never be harmed, period.^3^ But for all these philosophers, innocentism is more of a throwaway argumentative device than a view worth serious philosophical consideration.

I want to make the case that innocentism deserves more recognition than this, that it merits being taken seriously as a view one could reasonably endorse and defend. Taking innocentism seriously in this way requires, first, that we state the view, acknowledging its existence in the theoretical landscape; second, that we precisely state any objections we may have against it, rather than dismissing the view out of hand; third, that we allow it to be defended against objections, qualified, and refined (a privilege that unpopular views are often denied^4^). Taking unpopular philosophical views seriously in this way is helpful even if we do not endorse them. They might help us understand other people’s thinking, they often make for productive interlocutors, and engaging them can help us get a clearer view of the opposing positions we do take.

In what follows, I will discuss the main features of innocentism (section 2) and explain how it differs from retributive justice (section 3). I will then discuss how innocentism relates to widely-shared intuitions and opinions (section 4) and sketch a philosophical rationale that could be put forward in its support (section 5). In the final sections, I will situate innocentism in the theoretical landscape of animal ethics and explain why I think it is an addition worth being aware of (section 6). I will conclude by formulating some important challenges that innocentists face (section 7).

## Innocentism: The Basics

“We may be the only lawyers on earth whose clients are all innocent” reads a tagline of the Animal Legal Defense Fund, a non-profit organization for animal protection.^5^ The tagline is a pun. Attorneys defend their client’s *legal* innocence, and animals are *morally* innocent. Not being moral agents, they can literally do no wrong. And because animals are innocent, the tacit suggestion goes, we should care about what happens to them and support the non-profits that fight for them. This is just one of many manifestations of the idea that animals deserve particular moral concern in virtue of their innocence. Like “innocent children,” “innocent animals” is a familiar turn of phrase that emphasizes this special preciousness and value that must be protected from harm.

A more sinister manifestation of the same view can be found in the disturbingly common suggestion that scientific experiments (without informed consent) should be conducted on murderers and sex offenders rather than animals.^6^ This view is rightly taboo in official settings – in newspapers, in academic papers, and in politics – particularly because it conflicts with human rights and disregards human dignity. Speaking anecdotally, however, the view is very easy to find in more informal settings and crops up almost invariably when news of an animal testing scandal hits social media.

The view that looms in the background of such utterances is *innocentism*: We should care more about the innocent than the culpable, other things being equal. Culpability is moral responsibility for wrongs. So, in other words, the more numerous and serious the wrongs for which S is responsible, the less weight should be given to S’s interests. In the extreme, a moral transgressor becomes almost entirely morally insignificant and does not have to be seriously considered for their own sake anymore.

First things first. What do people mean when they call animals “innocent?” Clearly, the innocence at issue is *moral* innocence, as opposed to legal innocence, epistemic naiveté, or cultural constructs of innocence like virginity. Moral innocence appears to be a fundamentally negative property: It is *not* having done anything culpably wrong.^7^ The apparent problem with this purely negative notion is its radical overinclusivity – plants and rocks have not done anything wrong either, but we would not call them “innocent beings” like we do animals and small children. It seems that, to be innocent, one at least needs to be doing something.^8^ I suggest, therefore, that what people mean can be explicated as follows.

“Moral innocence” is a property characterized by two conditions (each necessary, jointly sufficient):

1. Agency condition: To be morally innocent, one needs to be an agent. Plants and lifeless objects, for example, cannot be innocent unless we assume they can act.^9^
2. No-culpability condition: To be morally innocent, one must not be morally culpable for any wrong.

This conception of moral innocence allows a clarification of who counts as morally innocent. They belong to one of two categories:

I. The contingently innocent: These are agents who can do wrong and bear moral responsibility, but are not actually culpable of any wrongs. This would be a moral saint, or a child right before becoming culpable for the first wrong of its life.
II. The necessarily innocent: These are agents who can do no wrong, in the sense of being incapable of committing wrongs or bearing responsibility for wrongs. Animals – those that act, anyway – belong in this group. Angels and gods would also belong here, assuming that they always want the good and are thus incapable of doing wrong. Animals and non-culpable human beings (like small children) are however the only innocent beings whose existence is uncontroversial.^10^

This conception, I believe, captures and clarifies the conceptual core of the everyday notion of innocence as it is commonly applied to small children and animals. However, what I have provided is a philosophical explication of the everyday notion, a conceptual simplification for clarity’s sake. It is not a dictionary definition whose extension matches perfectly with that of the everyday notion. For example, if the Terminator is an agent, but not a moral agent, the conception accepts him as an innocent being. This might be counterintuitive to say of a time-traveling killing machine. But some slight overinclusivity is a small price to pay for conceptual clarity, and so I suggest we should simply accept that the Terminator is innocent in this very specific, quasi-technical sense.

On the basis of the above conception, we can clarify three further points about moral innocence:

First, for some agents, it is possible to be innocent. With animals and non-culpable human beings, the innocent walk among us. Innocence is thus not an unattainable ideal or an exaggerated moralistic construct, at least not for everybody. It is a moral feature fully embodied by certain agents in the real world.

Second, moral agents are no longer morally innocent after they become culpable for their first wrong. Culpably committing a wrong is a contingent event, but it is extremely likely that it will happen sooner or later to any human moral agent. So, unfortunately but unsurprisingly, most humans lose their innocence shortly after beginning to bear moral responsibility.

Third, however, divergence from innocence comes in degrees, depending specifically on the number and the seriousness of one’s wrongs. Someone who is only culpable for a single lightweight wrong is still very close to being a saint, someone who is culpable of a litany of war crimes is far from being one. Notice, however, that being *less innocent* does not equal being *a worse person*. The latter is a yet more complex notion, sensitive to a person’s dispositions, their virtues and vices. One can become a better person over time, in part thanks to having experience in committing wrongs, but one cannot become more innocent. We can apologize for our wrongs, repent, make amends, and even be forgiven. This may well change whether others have standing to resent us for our wrongs or make claims for reparations, and it may affect how good we are as persons. But regret, repentance, and forgiveness do not undo culpability, and they cannot restore moral innocence.

With these clarifications in place, we can move on to innocentism’s normative claim, namely, that we should give preferable treatment to the innocent over the culpable. Most of us, I take it, value innocence in a somewhat passive way. We take pleasure in contemplating the moral innocence of small children and animals. Among other things, innocence is a crucial component of cuteness, which most of us enjoy.^11^ Innocentism, however, claims that our appreciation of innocence should go beyond the contemplative and into the practical. We should give special protection and care to the innocent, more so than to the culpable.

The most noteworthy upshot of this principle in animal ethics is that the interests of small children and animals should count *more* than the interests of human adults. Other things being equal, we should save a dog over a war criminal. Innocentism therefore clearly violates the Principle of Equal Consideration of Interests.^12^ It is an extremely rare case of a view that violates this principle on grounds that are intimately connected to species, but does so in favor of non-humans over most humans, not the other way around.^13^

Note, however, that this does not imply that we should always save animals and children over adult humans, all things considered. Innocentism does not *lexically* put animals above human adults, it only accords more weight to their interests. Four considerations are important to keep in mind:

First, a lot hinges on the number and strength of interests we take an animal and a human being to have. If, as some philosophers suggest, only human beings have an interest in continued life,^14^ or in liberty,^15^ or if human beings have a much stronger interest in these things,^16^ then human interests may often win even if the scales are tipped against them. Second, there can of course be cases in which more humans than animals are at stake, and human interests may then outweigh animal interests by sheer numbers. Third, not all human adults are equally affected by innocentism’s differential treatment. The interests of moral saints count as much as animal interests, those of generally well-acting people count only a little less, and those of extreme transgressors count a lot less. Just how steep this decline in significance should be is an open question. A reasonable version of innocentism should probably be lenient enough, counting the interests of generally well-acting people only slightly less than those of the innocent. Finally, innocentism does not determine which other moral principles should affect a weighing of interests. A lot is left open by the clause “other things being equal.” For example, innocentism is compatible with the view that one ought to care more about one’s loved ones than about strangers, or that one ought to care more about those suffering the gravest consequences, even if they are in the minority. It is even compatible with speciesism, in principle, though a speciesist innocentist would have to clarify how the two principles relate (should we save an innocent animal over a culpable human being – and just how culpable do they have to be?).

## Innocentism Versus Retributive Justice

By this point, some readers may wonder whether innocentism is not just a front for retributive justice. Is the reason why we should save a dog rather than a war criminal not simply that the war criminal *deserves* any suffering coming their way as punishment? Similarly, is the rationale for experimenting on murderers not that murderers *deserve* to suffer? And is the Animal Legal Defense Fund, in saying that their clients are all innocent, not simply stating that animals *do not deserve* their suffering?

Unfortunately, the few philosophers who mention innocentism do not distinguish it from retributive justice. Kagan and Jaquet both consider it an instance of retributivist thinking.^17^ I think that this obscures the matter. Saying that someone deserves punishment is *not* the same as saying that their interests matter less. To see the difference, consider the idea of retributive justice: A moral transgressor should receive a proportionate punishment for their wrongs (at least for certain kinds of wrongs). More specifically, Walen helpfully captures retributive justice in three principles.

1. that those who commit certain kinds of wrongful acts, paradigmatically serious crimes, morally deserve to suffer a proportionate punishment;
2. that it is intrinsically morally good – good without reference to any other goods that might arise – if some legitimate punisher gives them the punishment they deserve; and
3. that it is morally impermissible intentionally to punish the innocent or to inflict disproportionately large punishments on wrongdoers.^18^

The difference between innocentism and retributive justice can be seen in how an innocentist would diverge from these principles (though not necessarily contradicting them). In contrast to principle 1, an innocentist does not *assign* specific punishments to specific wrongs. The innocentist instead accords lesser weight to someone’s interests across the board, depending on the amount and seriousness of their culpable wrongs. In contrast to principle 2, an innocentist does not have to view the transgressor’s suffering as a morally *good* thing, just as less morally bad than the like suffering of an innocent being, other things being equal. And in contrast to principle 3, the innocentist does not call for *measured* responses to wrongs. The innocentist never stops caring less about the culpable, no matter how much suffering they have already endured.

Although innocentism is distinct from retributive justice, the two ideas are compatible and indeed are a natural fit. We can simultaneously think that transgressors deserve a certain measure of punishment – that deserved, legitimately inflicted suffering is good rather than bad – while also holding that we need not care so much about their suffering in case it is *not* deserved or legitimately inflicted. Thus, retributive justice paired with innocentism gives rise to a certain righteous coldness of heart. Notice that this coldness increases with the number and the weight of the wrongs at issue. Someone holding this view would still consider excessive punishment of a child outrageous, but would not care much about an excess in punishment for a war criminal.

Returning to the examples of innocentism I have introduced at the outset, I do not want to deny that they contain an element of retributive justice. But I think that the idea of innocentism plays a crucial role too. The Animal Legal Defense Fund, in emphasizing that “all our clients are innocent,” is not just saying that animal suffering is *not intrinsically good in one specific way*. The tagline also conveys that this suffering is *bad* and that it should *concern* us. So, what makes the tagline work is the idea that the innocent should receive particular protection. Similarly, retributive justice does not capture all that is going on in the example of the dog and the war criminal. Retributive justice calls for a *measured* response inflicted by a legitimate *punisher*. But there is no legitimate punisher in the burning building, and while we may find suffocating and burning alive too severe or too lax a punishment for the war criminal, it would be oddly specific to consider it *exactly* the right measure of punishment. At best, we can appeal to retributive justice to account for our enthusiasm for allowing the war criminal to suffer, but only innocentism can account for our lack of care whether the just measure of punishment is exceeded and whether a legitimate punisher inflicts it. The same can be said of those who call for experimentation on extreme transgressors rather than animals, who typically have no clear idea of just how much suffering they want to see inflicted, and by whom.

## Intuitions for Which Innocentism Can Account

At the outset of this paper, I have introduced innocentism as a view underlying certain common utterances in informal, non-academic debates about the treatment of animals. While some expressions of innocentism are dangerously insensitive to other ethical concerns – specifically the call to experiment on human beings without their consent – innocentism itself has a considerable intuitive pull for many people. No matter whether we agree with the view or share the intuitions it accounts for, we should acknowledge that for many people, there appears to be something this view captures.

Its intuitive pull is the reason why innocentism has been brought up in the literature at all. Kagan uses it as an intuitive counterexample to the Principle of Equal Consideration of Interests:

[…] isn’t it obvious that duration and intensity are the *only* two ways in which the significance of pains can differ morally?

Not at all. Suppose, for example, that you and I are both suffering in jail. We are equally miserable, and for an equally long time. But you are innocent, while I am being justly punished for some horrible crime. Can’t the fact that I *deserve* to be punished, while you do not, give us reason to think that the pain you are suffering should be *given more weight* than the pain that I am suffering? (Suppose someone could free one of us. Shouldn’t your suffering count for more than mine?)^19^

Although Kagan’s example does not distinguish legal from moral innocence, nor innocentism from retributive justice, it is innocentism that makes his example work. Retributive justice could explain why a culpable person’s suffering is a good thing, but not why their interests should be given less weight. Besides, even if two people, one deeply morally culpable and one innocent, were not incarcerated, but were stuck in a cave by accident, many of us would still save the innocent person first.

Note also that Kagan’s example works irrespective of how good the two people are. Say A is a serial killer who has recently seen the light, repented, and turned his life around. Not only is he now on his best behaviour at all times, but he has actually lost the dispositions that used to make him bad. A is currently exactly as good a person as B, a person who has never harmed anyone. The innocentist would not hesitate to save B from the cave rather than A, and I suspect many people agree. So what is at work here is not (only) a preference for good people over bad people, but a preference for the innocent.

Cross-species examples are also easy to construct, though innocentism’s intuitive pull is admittedly greatest when the human at issue is an extreme transgressor, as in the case of the dog and the war criminal. This however fits the fact that cross-species interest comparisons are difficult to begin with, since we must make assumptions about the kinds and strength of human and animal interests at stake. Thus, our intuitions are strongest when the human at issue is so deeply culpable as to be practically insignificant. Of course, if only extreme examples strike us as compelling, this might also be because our pro-animal judgments have to overcome our anti-animal prejudices.

On a more general level, innocentism can also account for various common views about the treatment of children. It explains why harming children is especially outrageous, and why they should receive special protection in armed conflicts and other emergency situations. To be certain, this might also be explained by children’s vulnerability. But if most people’s concern for children were rooted in concern for the vulnerable, one would also expect them to have concern for another famously vulnerable population, the incarcerated. Yet grave harms against the incarcerated are commonly ignored, even joked about. Concern for the vulnerable might be what we *should* have, but what many people *do* have, I suggest, is special concern for those they deem innocent.

There are other widespread intuitions for which innocentism can account, even though it is not alone in doing so. This shows that innocentism, despite its quirks, can be quite an agreeable view. For example, add to innocentism the assumption that power tends to increase culpability, because power corrupts, increases responsibility, and presents opportunities to wrong more individuals more seriously. Elected officials may even have special duties that *demand* moral wrongs, giving rise to the “problem of dirty hands.”^20^ The interests of the culpable powerful – think of politicians, crooked lawyers, the illicitly rich – therefore generally matter less than those of the powerless according to innocentism. And who in their right mind would save the crooked lawyer first? Also, consider that privilege in systems of oppression tends to increase culpability, as it is linked to power too, and you arrive at the intuitive view that the interests of the oppressed should concern us more than those of the privileged.

However, some of innocentism’s apparent upshots are disturbing. Most of us, including myself, abhor the idea of using humans, no matter how culpable, for experiments without informed consent. But while I believe that this idea typically arises from innocentist commitments, it is easy to avoid. To name just one counterargument, any relaxation of human rights protections would open the floodgates to abuse that harms innocent and generally well-acting people. So, an innocentist should defend human rights and not call for their undermining.

Innocentism also implies that we should generally prefer innocent animals over culpable human beings, other things being equal. While many people would presumably agree with this in extreme cases – such as the dog versus the war criminal – few people would accept this implication across the board. Indeed, a common objection against animal activists is that they value animals over people. Some of this rhetoric’s persuasiveness may be explained by anti-animal prejudice, but what if not all of it is? Surely we must take seriously that many find innocentism counterintuitive simply because it would have us systematically put animals above human adults.

In response, consider again the four points raised earlier: First, an innocentist does not *lexically* value animals over people. They only assign greater weight to the interests of innocent animals than to the like interests of culpable people. Second, human beings might have more, or stronger interests at stake. Third, the decline in moral deservingness with increasing culpability does not necessarily have to be very steep. Generally well-acting people may count just very slightly less than animals. And fourth, we can appeal to other moral principles, e.g. that one should prefer one’s loved ones over strangers, to defend a preference for certain human beings over certain animals in conflict cases. This gives the innocentist a lot of room for manoeuvre. We can value animals over human adults while still deciding in favor of humans in many cases.

Nevertheless, one must admit that giving greater weight to the interests of animals than to those of adult human beings would demand profound changes in the way humans and animals coexist, since children and animals outnumber human adults many times over. It would require changes that are at least as drastic as those required by the Principle of Equal Consideration of Interests, and then some. Innocentism would not only require that we stop most exploitation of animals for human purposes, but also that we use a much greater proportion of available resources for the benefit of animals than we currently do, even at the expense of human adults. For instance, a consistent innocentist might demand that we reduce funding for branches of medical research that benefit primarily human adults, and instead divert that funding to pediatric, veterinary, and ecological research that benefits the innocent. Even if we refuse to use human beings as models for animal bodies in veterinary research (out of respect for human rights), innocentism would indirectly require letting adult human beings suffer and die for the benefit of animals. Few people would be willing to accept this.

Consider, however, that innocentism only requires that we prefer children and animals over human adults in case their interests are truly at odds. But to some extent, what is good for adults is good for children and animals too, since all children and some animals need the support of human adults. Insofar as the good of adults, children, and animals are compatible and conflicts can be avoided, human adults have nothing to lose. Thus, thinking like an innocentist does not always force us adults to sacrifice our own interests, but it forces us to think from a disadvantaged position. This gives us an incentive to find win-win solutions and consider opportunity costs, rather than opting for convenient win-lose trade-offs at the expense of the innocent. An innocentist society would therefore not simply neglect research that benefits the culpable, but it would try to find ways to benefit primarily the innocent that also benefit the culpable. For example, research funding might be diverted to strongly One Health-oriented research designed to benefit both animal and human health. Even more importantly, an innocentist society would take more resources from the culpable powerful in the first place, increasing spending on *all* branches of science that benefit the powerless, be they animals, children, or adults.

An additional move open to the innocentist is to distinguish long-term ideals from immediate action-guidance. An ideal society might value the innocent over the culpable and use its resources accordingly. But this does not imply that all steps in this direction are desirable, because they might backfire or lead into strategic dead ends. Rather, endorsing the ideal commits us to carving out a path towards a future where the ideal is practicable. Similarly, an ideal society sends its children to school rather than to work, but this does not imply that societies should abolish child labor without considering the consequences for the children. Precautions need to be taken so that children do not simply starve due to lack of income, and instead receive the care and education they deserve. Similarly, endorsing innocentism does not imply that we cut medical research that only benefits adults without considering the consequences for existing research and healthcare systems. It implies that we strategically opt for a future where the innocent can safely be given preferable treatment over the culpable.

I hope to have shown in this section that innocentism can capture intuitions that many share, and that the deeply counterintuitive upshots it appears to have can be avoided. While I do not think that this alone makes the case for innocentism, I believe it moves it into the space of views one could seriously endorse. Next, let me explain why I also think that innocentism is a view one could reasonably defend on philosophical grounds.

## A Philosophical Rationale for Innocentism

Setting aside its intuitive pull, there is also something to be said for innocentism from a theoretical perspective. Although the rationale I want to sketch here will not suffice to convert many opponents to innocentism, I hope to show that innocentism admits of enough philosophical conceptualization and defense that philosophers sympathetic to the view could refine and defend it further, even if my case here is somewhat rough-and-ready.

First of all, innocentism is immune against objections that are typically directed against speciesism and other views that refuse to treat like interests alike. Perhaps the most prominent argument against speciesism is that species is morally irrelevant, and hence discriminating by species is arbitrary.^21^ One could object that innocentism is just reverse speciesism and suffers from the same problem. But unlike species, innocence is a moral notion, and it is not obvious that it should not affect how much we care about someone.

Another classic argument against speciesism is that it is a form of discrimination or oppression similar to, or intersecting with, the oppression of various human groups.^22^ The same cannot be said about innocentism. While oppression may well give rise to moral overburdening and moral dilemmas that the privileged do not face, the very fact of oppression should be taken into account when judging culpability. The oppressed may be forced to commit more wrongs, but they are all the less culpable for committing them. So innocentism does not disadvantage the already oppressed. As we saw in section 4 above, in fact the opposite is true, since it is privilege, not oppression, that tends to increase culpability.^23^

Second and more importantly, innocentism can be supported by philosophical arguments that resemble some well-known – and well-respected – arguments in ethics. Specifically, an innocentist can appeal to the value of innocence like a Kantian can appeal to the value of humanity. Even if we disagree with this appeal, we must admit that it has enough philosophical meat on the bone so that philosophers more sympathetic to it could refine and defend it further.

The value of moral innocence we might call a moral meta-value. By this I mean a value things have or lack in virtue of their relation to other moral values or norms. The most obvious example of a moral meta-value is the value that a person has in virtue of their morally valuable habits or traits, the value of the virtuous person. This value calls on us to admire these people, to praise them, and perhaps to make them our role models. Many of us would acknowledge this value because we already do emotionally relate to good people in this way – or at any rate, we ordinarily recognize that we should. This value seems plausible from the standpoint of ordinary moral phenomenology. What is more, it would seem difficult to accept the bindingness of moral norms without also accepting that observing these norms makes moral agents good in some way. If moral rules are binding, there is something valuable about those that act on them.

Kant, for one, also believed in another moral meta-value, namely, the value of humanity. Even a moral scoundrel has humanity, i.e. the *capacity* to be good, to determine their will from duty rather than inclination. Already in virtue of this capacity, human beings have a special kind of value. We may not *admire* or *praise* human beings for having the mere capacity to be good, but there is still something about them we can *approvingly recognize* or *respect*. According to Kant, the feeling of respect for humanity is merely a mediated form of respect for the moral law itself.^24^ So, once again, the idea roughly is that acceptance of the moral meta-value arises naturally from the acceptance of moral normativity. If moral rules are binding, there is something valuable about those who have the capacity to observe them.

The innocentist believes in yet another moral meta-value, the value of moral innocence. Like the value of the good person and the value of humanity, it is something we ordinarily recognize. We take spontaneous pleasure in contemplating moral innocence and the morally innocent, such as small children and animals, and this pleasure ties in naturally with a response of well-wishing. Where the value of the good person wins our admiration, and the value of humanity wins our respect, the value of innocence wins a kind of love or benevolence. We also, by the way, spontaneously mourn the loss of moral innocence. There is something lamentable about someone culpably committing a wrong, because they are irreversibly losing a bit of something we value about them. The value of moral innocence, then, seems plausible from the standpoint of ordinary moral phenomenology, just like the other meta-values. And where Kant argues that respect for humanity is mediated respect for the moral law, the innocentist can argue that love of innocence and the innocent is intimately connected to love of the good, to the recognition of moral normativity. If moral rules are binding, there is something valuable about those who have not violated them.

However, even if we accept that moral innocence has some special value, the innocentist must still explain why this value demands that we give preferential treatment to the innocent. Why is it not enough, say, that we passively enjoy this value when we see it embodied in the innocent, as we typically do? The innocentist’s answer could follow a similar path to one taken by some Kantians explaining why the value of humanity demands that we treat others with respect. According to Wood, values are linked to “expressive reasons for action”^25^:

[…] every action that is done for a reason (as distinct from being merely a response to an impulse) is based on regarding something as objectively *valuable*. When it is done for that kind of reason, the performance of the action is most fundamentally an expression of esteem for that value, and this expressive reason for performing the action is therefore the ground of any other reason we may have for performing it […].^26^

To put it another way, the actions we take express the values we endorse. Treating others with respect expresses reverence for the value of humanity, exploiting them expresses disdain for this value. In Wood’s view, the reason why we ought to treat others with respect, at the end of the day, is that only this expresses adequate appreciation for the value of humanity.

In parallel fashion, the innocentist can argue that giving preferential treatment to the innocent is the only adequate way to express reverence for the value of innocence. And since this value is so intimately bound up with moral normativity, the innocentist can argue that by honoring the innocent, we honor morality itself. The innocent embody the value of a life free from moral culpability, an ideal that is unattainable for us miserable human adults, but which we must recognize if we value the good. Conversely, those among us who fail to give greater weight to the innocent than the culpable express inadequate appreciation of morality, if not outright disdain for it. And after all, is there not something morally suspicious about someone who does not particularly love small children and animals?

These arguments gesture towards a philosophy of innocence that many may find moralistic, unconvincing, and obscure. But the same is true of many other views in ethics. What I hope to have shown here, anyway, is that innocentism admits of enough philosophical conceptualization and defense that it should be taken seriously as a view one could reasonably endorse. This is the case particularly because the notion of innocence is intertwined with notions of culpability, agency, value, normativity, and moral ideals, and because it does seem broadly plausible to say that innocence and the innocent are especially precious.

## Innocentism in the Landscape of Animal Ethics

Why is innocentism an interesting addition to the landscape in animal ethics? If being distinct, being endorsed by ordinary people, and admitting of philosophical conceptualization and defense is not enough, consider that innocentism shines an unfamiliar light on major approaches to animal ethics and the discussion about them.

First of all, innocentism is a provocative counterpart to anthropocentrism, the view that all human beings, and human beings alone, matter for their own sake. Paradigm cases of anthropocentrism are orthodox Kantianism^27^ and social contract theories^28^. Each in their own way, these views link moral deservingness to normative capabilities. An innocentist would agree that moral agency and moral standing are linked, but would claim that human adults matter *less* than animals, not more, due to moral agency. Innocentists endorse what Bernard Williams called a “Lutheran” form of human cosmic significance: significance as unique *lowering* below the rest of creation.^29^ Much more common is the “celebratory,” “Petrarchian” form, which uniquely exalts humans above all else.^30^ By endorsing a reasonable version of a Lutheran view, everyday innocentists make salient how philosophical discussions about potential links between moral agency and moral standing have been one-sided in favor of human-friendly, Petrarchian upshots, while animal-friendly, Lutheran moves have gone almost entirely unexplored.

Second, innocentism calls into question the egalitarianism that pervades animal ethics, and it does so from an unusual angle. If the first fifty years of academic animal ethics were boiled down to a slogan, it might be “Animals Matter No Less Than Humans.” To be sure, this slogan has been called into question. Some argue that not all animals are equal,^31^ that we have special obligations to particular animals,^32^ and simply that animals matter less than humans.^33^ Innocentism stands out in this discussion because it is the only view that gives preference to animals over most humans for reasons that are closely related to species.

From an innocentist standpoint, the egalitarianism that pervades animal ethics indeed appears objectionably self-righteous and complacent. Discussions in animal ethics are held exclusively among the culpable because the innocent cannot participate. We have every incentive to be biased in favor of the culpable. But how might the discussion go if there were moral saints in our world? What if they represented a sizable portion of the population? I do not find it obvious that we, the culpable, could defend treating a moral saint as if they were just another human, ignoring their superior moral value. At any rate, egalitarianism would not look like the progressive position in the discussion, but more like a compromise. But then, should we consider egalitarianism to be progressive just because we live in a world without saints?

Third, innocentism stands out in that it derives normative conclusions from a certain moral description of animals. Animal ethicists typically focus on the value of animals as experiencers of pleasure and pain,^34^ as subjects-of-a-life,^35^ as agents,^36^ and as beings capable of flourishing.^37^ The value of innocence stands out here because it is not the value of a non-moral natural property or capability of animals, but of a moral property. From an innocentist standpoint, the focus on animals as bearers of non-moral properties itself appears like an objectionable othering of animals. Why not treat animals as members of the moral community from the start, as we do with small children, acknowledging that they already have at least one moral property by default, namely, innocence? Thus, considering the view that the innocent should be preferred over the culpable helps to turn another dialectic of traditional animal ethics on its head.

In sum, no matter whether one endorses it, innocentism makes for a provocative and interesting interlocutor position that merits philosophical recognition and attention.

## Challenging Innocentism

I have argued that innocentism should be taken seriously as a view one could reasonably endorse in animal ethics. But taking a philosophical view seriously requires that one exposes it to any objections on may have. So, before I rest my case, let me conclude my discussion by formulating three challenges for innocentists. Whether they can be compellingly met, I am not certain, which is why I am not a committed innocentist.

The first challenge is to respond to arguments for egalitarianism. The most prominent theories in animal ethics do not make their egalitarian commitments arbitrarily. Specific lines of philosophical reasoning, e.g. about the role of impartiality in ethics^38^ or about the nature of moral rights,^39^ underpin these commitments. Innocentists must explain at which exact step of a given argument they disagree. For the moment, egalitarians have moral theories, while innocentists only have one weighing principle. Integrating it into existing theories or constructing a whole new approach to animal ethics is possible, but takes a lot of work.

The second challenge is to respond to debunking arguments. Casting out transgressors and giving special protection to children makes straightforward evolutionary sense.^40^ One could also try to debunk innocentism psychologically by appeal to moral foundations theory, arguing that it is merely an extreme expression of “ingroup” and “purity” values.^41^ Even philosophical debunking is an option: Innocentism might just be a confused variant of a preference for good people over bad people, which then irrationally spills over onto animals and small children despite the fact that they are morally neither good nor bad.^42^ In response, innocentists can attack the plausibility of the debunking accounts. Alternatively, they can turn the argument on its head and argue that, in fact, innocentism is *vindicated* by its functionality,^43^ by the fact that it gives philosophical expression to a set of moral intuitions that animal ethics usually neglects, or by its explaining something as rational that other philosophical approaches dismiss as irrational. But this discussion remains to be had in detail.

A third challenge is to explain how innocentism is compatible with a pluralistic society. If we do not mutually agree on what is right and wrong, and we care less about those we deem less innocent, this seems to favor a society of mutually indifferent and internally ultra-conformist moral communities. Should we really want to live in such a society? Of course, the innocentist can question whether we do not already live in such a society for better or worse. But to fully respond to the objection, the innocentist needs to situate their view within a larger political framework that determines just how much people in a liberal democracy need to care about each other. For instance, innocentism could be restricted to a purely personal principle, a part of what Rawls called one’s conception of the good, something separate from the principles of justice that should structure society.^44^ If innocentism is not so restricted, the innocentist needs to explain how the view does not undermine peace and liberal democracy under conditions of moral disagreement.

All of these challenges strike me as philosophically labor-intensive, but by no means impossible to meet. Few of us, the culpable, will be motivated to do the philosophical bidding of a cause that is manifestly against our interests, but we should keep in mind that one could. Even if we reject innocentism or reserve judgment, it is a view that deserves philosophical recognition.

## Footnotes

1 Shelly Kagan, “What’s Wrong With Speciesism?,” *Journal of Applied Philosophy* 33, No. 1 (2016): 1–21, p. 6. 10.1111/japp.12164.

2 François Jaquet, “What’s Wrong With Speciesism,” *Journal of Value Inquiry* (2020): 1–14, footnote 13. 10.1007/s10790-020-09781-y.

3 Tom Regan, *The Case for Animal Rights*. (Berkeley/Los Angeles: University of California Press), p. 287.

4 Keith Burgess-Jackson, “Taking Egoism Seriously,” *Ethical Theory and Moral Practice*, No. 16 (2012): 529–542, p. 537. 10.1007/s10677-012-9372-5.

5 Animal Legal Defense Fund, https://aldf.org/intellectual-property-terms-of-use/. Accessed March 15, 2022.

6 Notice that this call usually singles out offenders who have directly harmed children or other innocent beings. Not only the response is innocentist here, but also the choice of offender.

7 Another approach to innocence could define it in terms of “having a clean moral track record.” This would suggest that innocence requires moral agency, as one needs a moral track record to have a clean one. But because it is not applicable to moral non-agents, this cannot be the notion of moral innocence employed by people who care about the innocence of animals and small children. In principle, however, one could construct an anthropocentric version of innocentism on the basis of this more restrictive notion. I thank the anonymous reviewer for raising this point.

8 Regan alternatively draws the line at justice: “Those individuals can be properly viewed as innocent who can be treated unjustly“, Regan op. cit., p. 295. This seems doubtful. An anthropocentrist might affirm that animals are innocent beings while denying that we owe animals justice, while being perfectly competent in using the concept of innocence. However, what I will say about innocentism is compatible with Regan’s definition too.

9 As far as I can see, any philosophical conception of agency can be plugged into innocentism. However, I am interested in innocentism because it can capture certain intuitions about the value of innocent animals. So I will simply assume that, on some suitable conception, animals are agents, but not moral agents. If you like, think of Sebo’s “Perceptual Agency”; Jeff Sebo, “Agency and Moral Status,” *Journal of Moral Philosophy,* No. 14: 1–22. 10.1163/17455243-46810046.

10 For objections against the claim that animals are morally innocent, along with responses I agree with, see Regan op. cit., p. 294–97.

11 He (Michael) Jia, C. Whan Park, and Gratiana Pol. “Cuteness, nurturance, and implications for visual product design” in Rajeev Batra, Colleen Seifert, and Diann Brei (eds.), *The Psychology of Design: Creating Consumer Appeal* (New York: Routledge, 2016), 168–179.

12 Peter Singer, *Animal Liberation* (New York: Ecco Press, 2002); see also Kagan, *Whats Wrong*, p. 6.

13 There are other views that might, under current circumstances, give greater weight to the interests of animals. Egalitarian views might prioritize animals because we can increase their well-being more than the well-being of humans with the same amount of resources, prioritarian views might prioritize animals because they are comparatively worse-off (see Derek Parfit, *Equality or Priority? The Lindley Lecture* (University of Kansas, 1995); Shelly Kagan, *How to Count Animals, More or Less* (Oxford: Oxford University Press). 10.1093/oso/9780198829676.001.0001). But animals are favoured by these views because they are species-blind. By contrast, innocentism favours animals because animals are innocent, a property they have due to their species-dependent inability to carry moral responsibility.

14 Peter Singer, *Practical Ethics* (Cambridge: Cambridge University Press, 2011). 10.1017/CBO9780511975950.

15 Alasdair Cochrane, *Animal Rights Without Liberation: Applied Ethics and Human Obligations* (New York: Columbia University Press, 2012).

16 Robert Garner, *A Theory of Justice for Animals: Animal Rights in a Nonideal World* (Oxford: Oxford University Press, 2013). 10.1093/acprof:oso/9780199936311.001.0001.

17 Kagan *What’s Wrong* op. cit.; Jaquet op. cit.

18 Alec Walen, “Retributive justice” in Edward N. Zalta (ed.), *The Stanford Encyclopedia of Philosophy (Summer 2021 Edition)*. https://plato.stanford.edu/entries/justice-retributive/. Accessed March 15 2022.

19 Kagan *What’s Wrong* op. cit., p. 6.

20 Michael Walzer, “Political Action: The Problem of Dirty Hands,” *Philosophy & Public Affairs* No. 2 (1973): 160–180.

21 Jaquet op. cit.; Singer *Practical Ethics* op. cit.

22 Singer *Animal Liberation* op. cit.; Adams, Carol J., *The Sexual Politics of Meat: A Feminist-Vegetarian Critical Theory* (New York: Bloomsbury Academic, 2015).

23 Thanks to Emnée van der Brandeler for helpful discussions on this point.

24 Ernesto V. Garcia, “A new look at Kantian respect for persons,” *Kant Yearbook* No. 4 (2012): 69–89. 10.1515/kantyb.2012.69.

25 Allen W. Wood, *Kant’s Ethical Thought* (Cambridge: Cambridge University Press, 1999), p. 141. 10.1017/CBO9781139173254.

26 Wood op. cit., p. 142.

27 See, for instance, Heike Baranzke, “Tierethik, Tiernatur und Moralanthropologie im Kontext von § 17, Tugendlehre,“ *Kant-Studien* No. 96 (2005): 336–363. 10.1515/kant.2005.96.3.336; Barbara Herman, “We Are Not Alone: A Place for Animals in Kant’s Ethics,” in Eric Watkins (ed.), *Kant on Persons and Agency* (Cambridge: Cambridge University Press, 2018), 174–191. 10.1017/9781316856529.011.

28 Peter Carruthers, *The Animals Issue: Moral Theory in Practice* (Cambridge: Cambridge University Press, 2002). 10.1017/CBO9780511597961.

29 Bernard Williams, “The Human Prejudice” in Adrian W. Moore (ed.), *Philosophy as a Humanistic Discipline* (Princeton, NJ: Princeton University Press, 2006), 135–154.

30 Ibid, 136–137.

31 Clare Palmer, *Animal Ethics in Context* (New York: Columbia University Press, 2010); Sue Donaldson and Will Kymlicka, *Zoopolis: A Political Theory of Animal Rights* (New York: Oxford University Press, 2011); Shelly Kagan, *How to Count*.

32 Palmer op. cit.; Keith Burgess-Jackson, “Doing Right By Our Animal Companions,” *The Journal of Ethics* No. 2 (1998): 159–185.

33 Garner op. cit.; Kagan *How to Count*.

34 Singer *Animal Liberation* op. cit.; Singer *Practical Ethics* op. cit.

35 Regan op. cit.

36 Christine M. Korsgaard, *Fellow Creatures: Our Obligations to the Other Animals*. (Oxford: Oxford University Press, 2018). 10.1093/oso/9780198753858.003.0008.

37 Martha C. Nussbaum, *Frontiers of Justice: Disability, Nationality, Species Membership* (Cambridge, MA: Belknap Press of Harvard University Press, 2007). 10.2307/j.ctv1c7zftw.

38 Singer *Practical Ethics* op. cit.

39 Regan op. cit.

40 See Jaquet op. cit.

41 See Jonathan Haidt and Jesse Graham, “When Morality Opposes Justice: Conservatives Have Moral Intuitions That Liberals May Not Recognize,” *Social Justice Research* No. 20 (2007): 98–116. 10.1007/s11211-007-0034-z.

42 I thank the anonymous reviewer for this suggestion.

43 See Matthieu Queloz, “How Genealogies Can Affect the Space of Reasons,” *Synthese* No. 197 (2018): 2005–2027. 10.1007/s11229-018-1777-9.

44 See John Rawls, *A Theory of Justice (Revised Edition)* (Cambridge, MA: Belknap Press of Harvard University Press, 1999), esp. p.491–496.

